# Trends in Pediatric Malpractice Claims at a Tertiary Children’s Hospital

**DOI:** 10.3390/healthcare13162051

**Published:** 2025-08-19

**Authors:** Beatrice Defraia, Simone Faccioli, Emanuele Gori, Barbara Gualco, Rossella Grifoni, Massimo Pacitti, Fortuna Pierro, Ilaria Lombardi, Vilma Pinchi, Martina Focardi

**Affiliations:** 1 Forensic Medical Sciences, Department of Health Science, University of Florence, 50100 Florence, Italy; simone.faccioli@studiofaccioli.eu (S.F.); barbara.gualco@unifi.it (B.G.); rossella.grifoni@unifi.it (R.G.); vilma.pinchi@unifi.it (V.P.); martina.focardi@unifi.it (M.F.); 2 General Direction of Meyer Children’s Hospital, University of Florence, 50121 Florence, Italy; direzione.sanitaria@meyer.it (E.G.); massimo.pacitti@meyer.it (M.P.); fortuna.pierro@meyer.it (F.P.); ilaria.lombardi@meyer.it (I.L.)

**Keywords:** pediatric claims, malpractice claims, medico-legal cases, children’s injury, evaluation of pediatric damage, errors in pediatrics

## Abstract

**Background**: Examining medico-legal cases within hospitals aids in identifying care-related problems, facilitating necessary corrections and emphasizing successful preventive measures. The case of Meyer Children’s Hospital is particularly noteworthy as it offers insights into the evolution of litigation in regard to a tertiary pediatric hospital. **Methods**: The study sample comprised 158 malpractice claims received by Meyer Children’s Hospital from 1 January 2010 to 31 December 2023, which were managed by the Claims Management Committee (CMC) responsible for civil liability within the hospital. In this observational retrospective study, the following variables were analyzed: (1) The characteristics of the patients (age–sex) and the manner in which they interacted with the hospital, ultimately resulting in the compensation claim (method of access, area of specialty, outcomes based on the International Classification of Patient Safety (ICPS)). (2) Medico-legal factors: the details of the compensation claim, the significant issues noted in various cases, and the findings of the medico-legal inquiry conducted by the CMC. In cases of ADR (Alternative Dispute Resolution), we evaluated the nature of the procedure, the results, and the amount of compensation awarded. **Results**: We conducted a descriptive statistical analysis to delineate the trend of claims and identify specific deficiencies within structures or departments over time. Invasive procedures and surgical operations were identified as the leading causes of accidents, resulting in heightened mortality rates and serious injuries. The most common errors observed were diagnostic and therapeutic. **Conclusions**: The data that emerged highlighted a low rate of claims (11.28/year) and a low claim/service ratio (0.0002%), suggesting a high level of safety of patient care at the hospital. The acceptance rate (32%), the percentage of rejected cases (48/158~30% of total, or 48/99~49% of resolved claims), the average compensation (EUR 68,312), and the percentage of cases (92%) with judicial opinions consistent with those of the CMC indicate a tendency to pursue exploratory compensation requests and the effectiveness of CMC’s activity. Meanwhile, the predominant error types (surgical and diagnostic) are in accordance with national and international data. Finally, the scarcity of disputes concerning informed consent reflects the impressive effectiveness of the communication strategies utilized by the pediatricians at this center.

## 1. Introduction

Few studies have concentrated on medical malpractice claims involving children, and most of these studies are case reports related to pediatricians [1,2,3,4,5,6,7,8].

The incidence of child malpractice cases presented in court, the judicial proceedings, the healthcare professionals responsible for these malpractices, the compensation granted, and the causes of these malpractices vary from one nation to another [9,10,11]. In a study analyzing 214,226 closed medical malpractice claims made in the USA from 1985 to 2005, pediatricians ranked tenth among 28 specialties regarding the number of closed claims, representing 2.97% of these claims [12]. Furthermore, pediatricians ranked 16th in terms of the rate of indemnity payments. According to the Supreme Court of Japan, in 2003, there were 1019 newly accepted lawsuits concerning medical malpractice, of which only 21 were filed against pediatricians. Additionally, for every 100 obstetricians/gynecologists, there were 0.62–0.91 lawsuits per year, whereas for every 100 pediatricians, there were 0.18 lawsuits per year in Japan. In a study conducted in Turkey on malpractice claims made between 1995 and 1999, pediatricians ranked fifth among other specialties, accounting for 4.6% of all malpractice claims [13]. Finally, a study conducted in France and reported by the Sou Médical-groupe MASCF insurance company over a 5-year period (2003–2007) showed that the average annual incidence of malpractice claims was 0.8/100 pediatricians and that they were more frequent (41%), with more severe outcomes, for children younger than 2 years of age (52% deaths or major injuries) [14].

The evidence base for pediatric medical malpractice claims in Italy is severely limited. A single study on neonatal claims [15] provided minimal data and focused only on the neonatal period rather than the broader pediatric population. The absence of comprehensive pediatric malpractice registries or systematic data collection in Italy represents a significant knowledge gap.

The available general malpractice data for Italy [16] provide context but lack pediatric-specific breakdowns. In addition, international comparisons, while informative, may not be directly applicable due to the different healthcare systems, legal frameworks, and cultural contexts.

Research has revealed that medical malpractice related to children primarily occurs in emergency departments, with general practitioners, pediatricians, orthopedists, and general surgeons being the main professionals implicated [17].

The framework of the Italian National Health System (NHS) resembles the Beveridge model created by the British NHS. Similarly to the British NHS, the Italian population receives healthcare coverage that is primarily funded and provided by the government through taxation. Consequently, the National Health Service (NHS) has been burdened by rising insurance premiums and healthcare expenses.

Analyzing the compensation provided for damages in Italy in relation to other nations is challenging due to considerable variations in the legal definitions, processes, and criteria used to award damages. The absence of a standardized or easily comparable framework across different countries complicates direct comparisons. Additionally, in Italy, the term “biological damage” (danno biologico) denotes the deterioration of an individual’s physical or mental health as a result of injury and is compensated separately from lost income. This notion is distinctive to Italian jurisprudence and contrasts with the approach to personal injury used in numerous other countries, where compensation typically emphasizes lost wages and medical costs.

The analysis of the Medmal report relating to the years 2004–2011, based on a sample of 95 Italian public health hospitals, shows an increase in compensation requests, which increased from a minimum of 2289 in 2004 to 3621 in 2011; a peak was reached in 2009, with 6290 requests in a single year [18].

The estimates presented by the National Association of Insurance Companies (ANIA) show a clear increase in claims reported not only by hospitals (6345 claims reported in 1994 and 21,476 in 2009) but also by doctors (3222 in 1994 and 12,559 in 2009).

Moreover, there has been a notable rise in the average cost of claims, which is particularly pronounced in cases that are generally more complex and demanding, necessitating a longer resolution period. Prior to 2009, in Italy, most civil liability disputes were adjudicated by an ordinary judge, despite the existence of various forms of Alternative Dispute Resolution (ADR) in Italian legislation that could potentially be more cost effective.

In 2009, the Council of Tuscany (one of the 21 regions of Italy, of which Florence is the chief town) began an experiment aimed at enforcing ADR, particularly the out-of-court settlement of health disputes. The Council established Claims Management Committees (CMCs) for civil liability in the Tuscan Health Service, with the primary objective of reducing recourse to the courts and litigation costs. This experiment was conducted for risk management and cost-saving purposes. The CMC settlement provides that damages are compensated directly by the hospital, removing the cost of insurance management from the claim. In fact, based on the documentation obtained from the Directorate-General, regional annual spending on insurance policies was around EUR 50 million, compared to an annual expenditure of compensation claims not exceeding EUR 5–6 million [19].

The Tuscan initiative aims to encourage extrajudicial conciliation, primarily due to challenges and the high cost of medical malpractice insurance. The CMC trial represents a form of Alternative Dispute Resolution (ADR).

Meyer Children’s Hospital operates within the national health system of Tuscany and has a decentralized management structure. The CMC addresses claims through a structured process involving a team that includes legal and administrative officials, risk management experts, and forensic medicine specialists.

To ensure transparency and expedite claim resolutions in Tuscany, the CMC establishes a database for claims, reviews medical records, conducts initial analyses within 15 days, and seeks expert opinions. Compensation is funded by the Tuscan region through the Regional Health Fund.

Meyer Children’s Hospital is a specialized pediatric facility that provides high-quality care for complex cases. It works with top centers such as The Children’s Hospital of Philadelphia and Boston Children’s Hospital. The hospital is involved in European Reference Networks for rare diseases, has three Centers of Excellence, and has received various accreditations. Since 2010, it has provided an average of 532,888 services annually. From 2010 to 2023, an average of 5930 surgical interventions (all performed in the operating room, with 70% hospitalized and 30% in day hospital) were performed per year. In addition, an average of 38,754 day hospital cycles were opened and 35,221 children were admitted to the emergency room [20,21,22].

At the Meyer Children’s Hospital, the CMC was established through resolution no. 242 dated 22 December 2009, which was subsequently renewed by resolution no. 122 on 4 March 2020 [23]. This resolution specified that, due to a lack of forensic doctors and a loss adjuster among the hospital staff, it was necessary to establish specific agreements or freelance contracts to engage these professionals. Both the forensic doctors and the loss adjuster were contracted by Meyer Hospital from another tertiary facility and possessed considerable experience in medical liability and damage compensation. They have previously served as insurance advisors and are often appointed by civil and criminal courts in cases related to health claims. The consultants within the CMC reconstruct the course of the clinical case, highlight the critical issues attributable to the healthcare professionals, and assess the consequences these have had on the patients’ health, whom they examine with the support of specialists in the relevant field when necessary. They then proceed to evaluate the compensation proposal and the risks associated with the potential judicial development of the compensation claim. Considering that the medical malpractice system in Italy differs from that in the USA, France, and Turkey, our goal was to analyze pediatric malpractice claims within a tertiary pediatric hospital in Italy. We sought to compare these claims with national data to improve our understanding of medical errors in pediatrics and to identify critical areas for interventions that could enhance the safety of pediatric care.

## 2. Materials and Methods

This is a retrospective, monocentric study. The authors analyzed all claims involving patients from 1 January 2010 to 31 December 2023. Permission was obtained from the data protection officer and general manager [24].

From all (192) compensation requests, 34 were excluded as they could not be traced back to facts pertaining to medical liability.

All cases, suitably anonymized, were input into a specialized database comprising a digital spreadsheet. The recorded variables and criteria established for documenting each type of variable are outlined below:

- **Unique identification code of the claim**: implemented to ensure traceability within the database.
- **Age and sex of the injured patient.**
- **Event date.**
- **Method of admission to the hospital**: ordinary/urgent/emergency. In cases of department transfers, the severity assigned to being admitted to the department in which the incident that prompted the compensation request occurred was noted. Clinical improvement or deterioration during hospitalization was deemed irrelevant to the initial access method.
- **Type of setting** to which the issue raised by the applicant pertains. The categories identified included the emergency department, hospitalization (which encompasses short-term hospitalizations, such as day and week hospitals), outpatient services, consultation, teleconsultation, and laboratory services.
- **Specialist area associated with the incident that led to the compensation request.**
- **Nature of the issue for which compensation is sought.**

Errors were classified as follows:

- Surgical errors, which concerned invasive procedures, including endoscopic treatments and outpatient surgical procedures;
- Therapeutic errors, which involved the administration of drugs or other non-invasive forms of therapy;
- Diagnostic errors, which pertained to the recognition of a pathological condition, whether through laboratory or instrumental examinations, or by direct patient observation.

For these three types of errors, the subcategories “commissive” (when the error consisted of performing an action that should not have been performed) and “omissive” (when the error involved omitting an action that should have been performed) were adopted.

Additionally, errors related to information and consent (where it was not possible to demonstrate the acquisition of valid consent following comprehensive and proper information), organizational deficiencies (when the error resulted from inadequacies in internal procedures within the institution, such as sterilization or transport procedures), and device defects (including cases in which the defect could potentially be attributed to the manufacturer) were also categorized:

- **Date of formulation of the first compensation request**;
- **Date of closure of the litigation;**
- **Type of damage referred to in the complaint;**
- **Technical consultancy on behalf of the party;**
- **Involvement of third parties and agreements with other hospitals;**
- **Medico-legal evaluation of the damage;**
- **Path of the claim:** for each compensation claim, both the type and number of judicial and extrajudicial steps undertaken were considered, including the sequence in which these steps occurred;
- **Claim outcome:** the compensation claims were categorized according to the final outcome established either in extrajudicial or judicial proceedings:
  1. Accepted if, at the conclusion of the litigation process, any sum of compensation was granted (blue in the columns);
  2. Rejected if, at the conclusion of the litigation process, no compensation was granted (red in the columns);
  3. In progress if the case is actively developing, irrespective of the stage attained and any significant inclination towards acceptance or rejection (purple in the columns);
  4. Without a follow-up if, despite repeated reminders, the claimant failed to respond to Meyer’s requests for documentation, for undergoing a medicolegal examination, or for providing clarification. This lack of cooperation prevents Meyer from proceeding further with the processing of the case. The time required for the CMC to classify a case as closed without further action is generally 18 months (green in the columns).
- **Official technical consultancy (OTC).** We observed whether one or more OTCs were conducted for each request, at what stage in the process they were organized, and whether they provided evidence for the rejection or acceptance of the compensation request.
- **Definition of the amount of compensation.**
- **Recurrence of rare disease.** All subjects were considered to have a rare disease if there was documentary evidence of them being affected by pathological conditions, or variants thereof, whose recurrence in the literature is reliably attested to be less than or equal to 1 in 2000. Subjects were classified as affected even if the rare disease in question was not related to the issue that led to the compensation claim.
- **ICPS results**. In instances where Meyer Children’s Hospital was acknowledged as liable, the damage resulting from such liability has been categorized according to the International Classification of Patient Safety. The World Health Organization’s International Classification for Patient Safety (ICPS) identified incident characteristics, patient outcome (none; mild, moderate; severe; death), and their contributing factors in cases involving complications of medical or surgical care [25].

This classification identifies the following levels:

- None—Patient outcome is not symptomatic, no symptoms are detected, and no treatment is required.
- Mild—Patient outcome is symptomatic, symptoms are mild, the loss of function or harm is minimal or intermediate but short term, and no or minimal intervention (e.g., extra observation, investigation, review or minor treatment) is required.
- Moderate—Patient outcome is symptomatic, intervention is required (e g., additional operative procedure; additional therapeutic treatment), an increased length of stay is required, or permanent or long-term harm or loss of function is caused.
- Severe—Patient outcome is symptomatic, life-saving intervention or major surgical/medical intervention is required, and a shortened life expectancy, major permanent or long-term harm, or loss of function is caused.
- Death—When balancing the probabilities, death was caused or hastened in the short term by the incident.

The findings were examined using descriptive statistics.

All of the variables and parameters are summarized in Table 1.

Due to the descriptive nature of the statistical analyses performed, the sample size and the absence of comparable case series in the literature, we chose not to exclude cases that were incomplete in one or two variables from the sample. In each analysis, we specified the number of cases in the sample that actually provided the information considered.

## 3. Results

### 3.1. Numbers of Claims per Year

**Figure 1:**
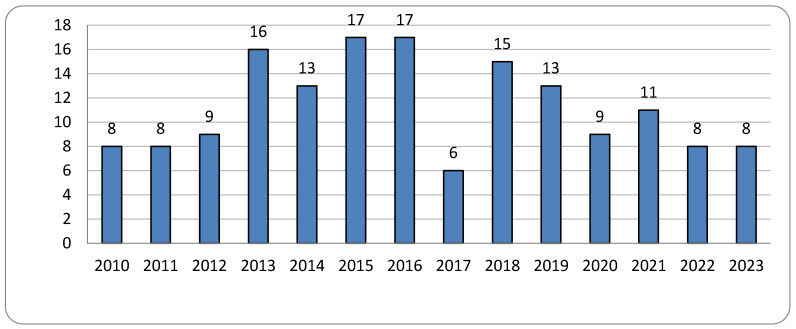
Number of claims per year.

A total of 158 claims of medical malpractice were documented (56 were resolved in one step by the CMC). The distribution of claims across the different years analyzed is illustrated in the subsequent graph (Figure 1). The average number of claims each year is 11.28.

### 3.2. Outcome of Claims

**Figure 2:**
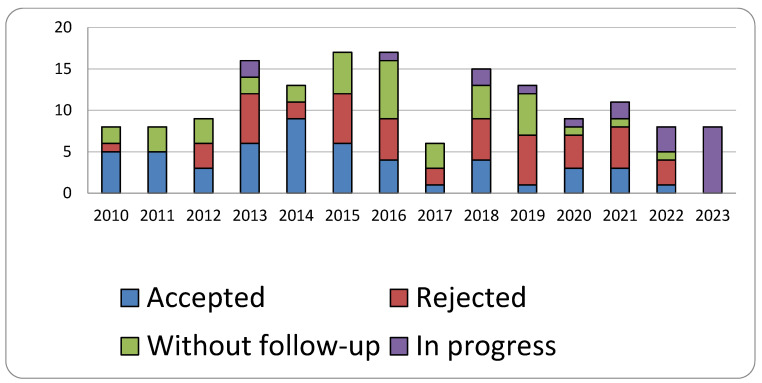
Outcome of claims per year.

Of the 158 claims, 51 have been settled and accepted, 48 have been settled and rejected, 20 are currently in the process of being settled, and 39 have not been pursued further. The progression of claims based on the year of filing is depicted in the graph below (Figure 2):

### 3.3. Age and Sex of Patients

Information regarding the ages of the injured individuals at the time of the incident was available for 155 of the 158 cases.

Five cases addressed matters pertaining to prenatal monitoring, and two compensation claims were filed by the parents of a child for themselves. These claims, due to specific factors pertaining to individual compensation claims, were deemed inappropriate and inconsistent when compared with the other cases. Consequently, these seven cases were excluded and limited to the evaluations regarding sex and age.

**Figure 3:**
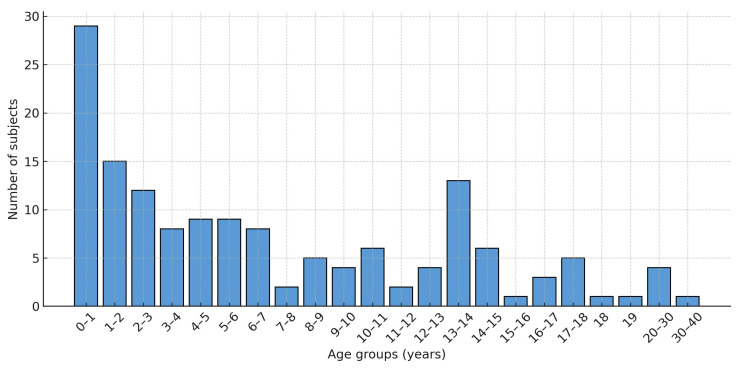
Distribution of claims per age.

The average age, including patients > 18 years old (among the seven subjects aged 18 years or older included in the case study, there were individuals afflicted by a rare disease who have received treatment at the Meyer Hospital since childhood; however, the damage manifested between the ages of 18 and 30), was 7.10 years, with a median age of 5.13. The distribution across the different age categories aligns with the trend illustrated in the following graph (Figure 3).

**Figure 4:**
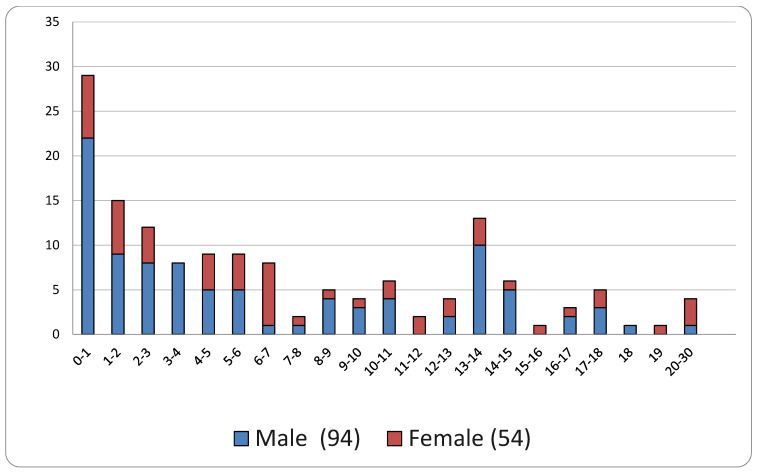
Distributions of claims per age and sex.

A graphic distribution of the patients by sex (blue in the columns for male and red in the columns for female) is presented below (Figure 4).

### 3.4. Claim Management Process

The timing of dispute resolution was initially examined in the claims management process. The analysis focused exclusively on cases resolved through either the acceptance or rejection of the compensation request, as the overall duration of dispute resolution in ongoing cases remains indeterminate. For the 99 cases that were resolved with either acceptance or rejection, an analysis encompassing two separate time intervals was performed: the first interval spanned from the date of the event that prompted the compensation request to the submission of the request itself; the second interval extended from the date on which the request was submitted to its final resolution.

**Figure 5:**
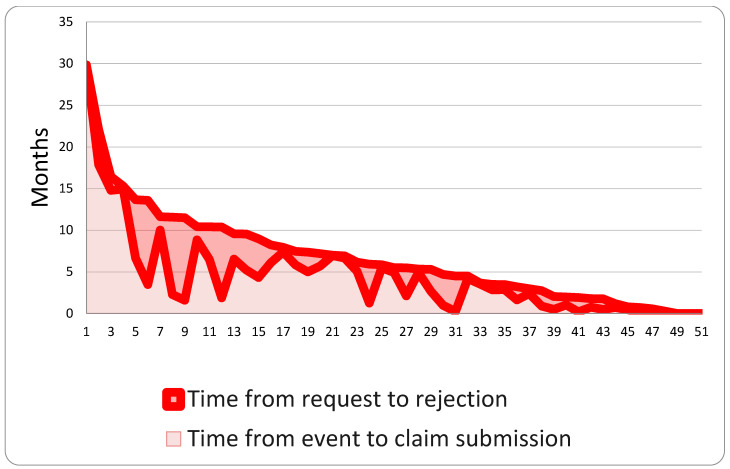
Time between claim submission and its rejection.

Compensation claims were submitted an average of 1307 days (approximately 3.6 years) after the event, ranging from 24 days to 10,888 days. The median follow-up period was 973 days. Once requested, claims took an average of 1017 days (approximately 2.8 years) to resolve, with a minimum of 5 days and a maximum of 4299 days. The median resolution time was 675 days. Rejected claims were submitted, on average, 4.76 years after the event and took approximately 2.37 years to resolve, with the process taking an average time of 7.13 years (Figure 5).

**Figure 6:**
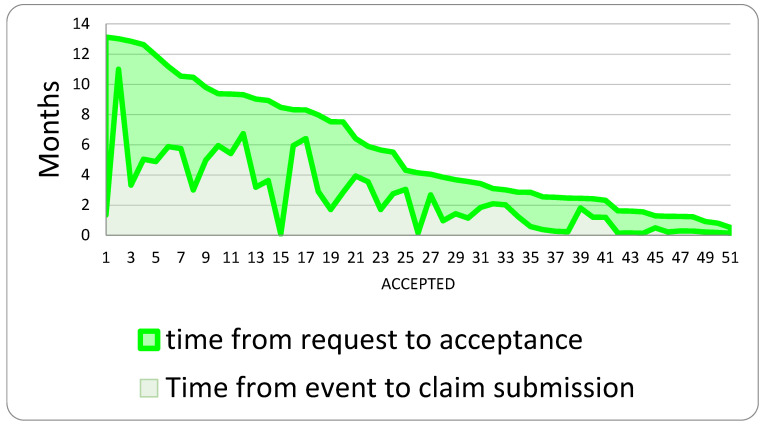
Time between claim submission and its acceptance.

Accepted claims are usually submitted approximately 2.46 years after the event and take an average of 3.17 years to resolve. On average, there are 5.64 years between the event and the denial of a claim (Figure 6).

**Figure 7:**
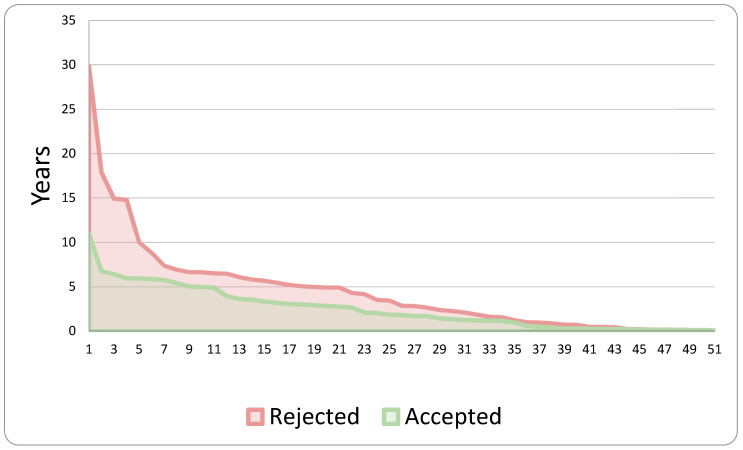
Years between event occurrence and initiation of the claim.

**Figure 8:**
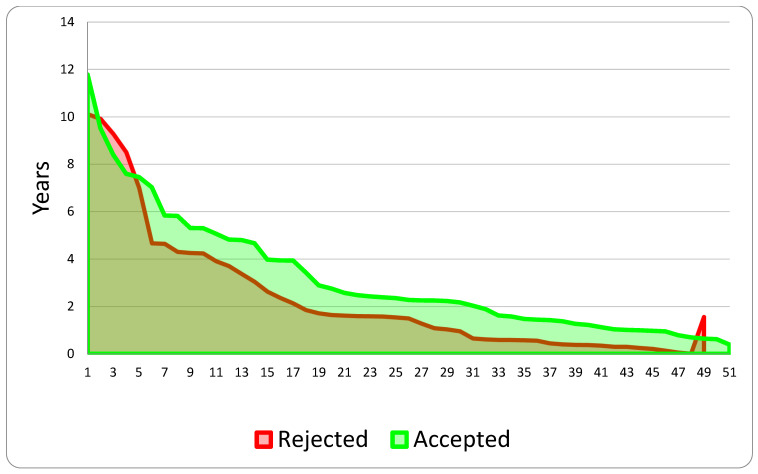
Years between the claim and its definition.

The analysis of the two subgroups concerning the two separate phases of claim development examined (first, the interval between the occurrence and submission of the compensation request; second, the duration between the submission and establishment of the aforementioned request) is illustrated in the subsequent two graphs (Figure 7 and Figure 8).

The median duration of the 56 cases that were resolved in only one step by the CMC was approximately 590 days, which means that the median implementation time after judicial proceedings was 567 days for acceptance and 275 days for rejection.

### 3.5. Trend of the Claim with ADR and Judicial

**Figure 9:**
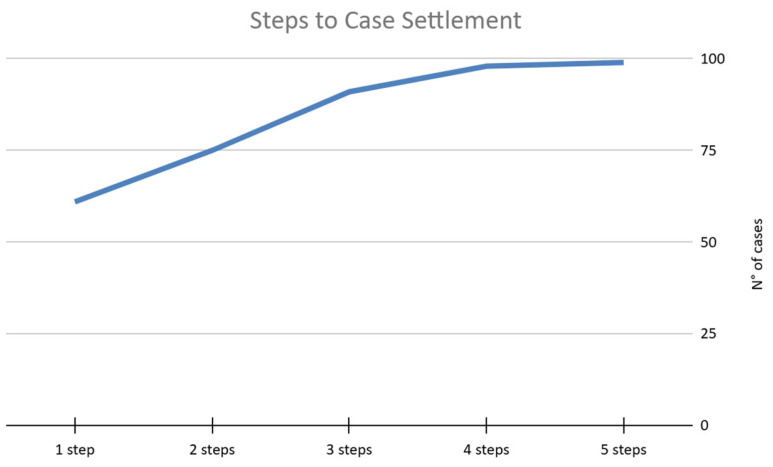
Trend in claims and their steps.

In total, among the 99 claims that were resolved (either through the acceptance or rejection of the compensation proposal), 61 cases were resolved in one step (56 were resolved via CMC evaluation alone and 5 were resolved via judicial evaluation or Article 696 bis, a preventive technical assessment regulated by the Italian Civil Law); 14 were resolved in two steps (all were resolved via CMC evaluation and 12 mediations and 5 judicial evaluations or Article 696 bis); 16 were resolved in three steps (all were resolved via CMC evaluation and 4 mediations and 9 judicial evaluations or Article 696 bis), 7 were resolved in four steps (all were resolved via CMC evaluation, some with mediation and all with judicial or the 696 article), and a single case required five steps (this case was solved via multiple CMC evaluations after mediation and judicial and 696 evaluation) (Figure 9). We intended “step” to denote a phase of the medico-legal evaluation process between extrajudicial and judicial. It is noteworthy that, of the 61 cases resolved in a single step, 56 were managed through the CMC. In addition, 7 of the 14 cases resolved in two steps were defined through out-of-court management by the CMC, followed by mediation. Of the 99 cases, seven were resolved following a judicial procedure alternative to the first-instance civil proceedings (Article 696 bis) (the compensation claim was rejected in six cases, while one was upheld), whereas 14 were resolved on the basis of a first- or second-instance court judgment (half were upheld and half were rejected).

Of the 99 cases examined, CMC anticipated the presence of liability in 41 instances: 29 were upheld directly, 8 were upheld following mediation (either prior to and/or subsequent to direct management by the CMC), and the remaining 4 were resolved through judicial proceedings (first or second instance), primarily due to disputes concerning the quantification of damages, which were ultimately adjudicated in a manner favorable to the quantification proposed by Meyer.

In 51 cases, the Meyer CMC anticipated the absence of liability: 27 were directly and effectively dismissed by the CMC, 6 were dismissed following mediation (either prior to and/or subsequent to direct management by the CMC), and 11 were dismissed through judicial proceedings (six under Article 696 bis, four through ordinary proceedings, and one on appeal).

Overall, the liability determinations forecast by Meyer in 41 cases were confirmed in all instances, albeit occasionally following additional procedural steps concerning the quantification of damages; in such instances, the final determinations were nevertheless more closely aligned with Meyer’s assessment than with the claimant’s request.

Of the 51 cases in which rejection was forecast, dismissal was effectively confirmed in 44.

Accordingly, in 99 cases, the CMC’s forecast aligned with the final outcome in 92 instances, corresponding to an agreement rate exceeding 92%.

Meyer’s assessment of no liability was overturned in seven cases (two under Article 696 bis and five in ordinary proceedings): in one case, the court judgment diverged from the court-appointed expert’s opinion; in one case, minimal damages were awarded relative to the claim; and in one case, minimal compensation was granted to avoid the continuation of the judicial process.

### 3.6. Clinical Setting and Type of Recovery

Regarding the establishment of the requests, in relation to the severity upon admission and the context in which the service was rendered, the initial sample comprised 157 compensation requests. This number decreased to 151 because, in six instances (four of which remained unanswered, and two of which were rejected), the compensation request submitted was significantly incomplete and vague, or the incidents occurred outside of a recognizable care setting). Of these 151 cases, 105 pertained to incidents that took place during hospitalization, 5 admissions were classified as emergency, 42 were classified as urgent, and the remaining 58 were categorized as ordinary admissions; 18 pertained to services rendered on an outpatient basis following ordinary admission; 17 involved emergency room services, 1 of which was classified as an emergency, while the other 16 were urgent; 9 involved consultations, with 7 being urgent, 1 classified as an emergency, and 1 as ordinary; and 2 involved laboratory services, with 1 being urgent and 1 ordinary.

**Figure 10:**
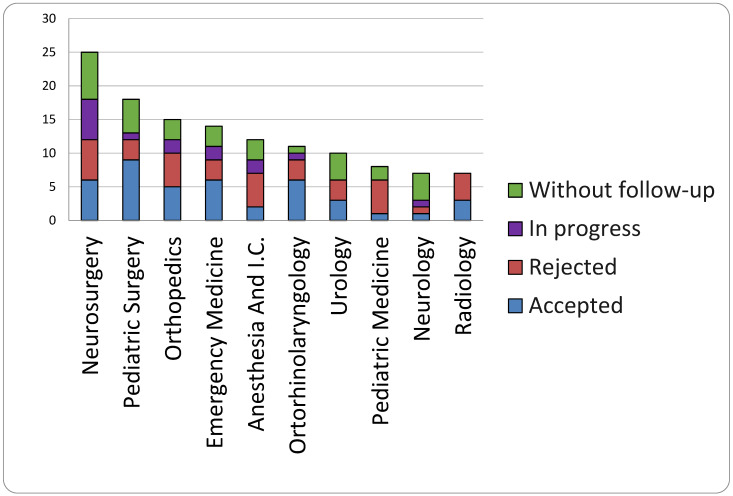
The ten specialties that have more claims.

The graph (Figure 10) shows the ten specialties with the most claims. The five leading specialties based on the number of claims represented over 50 percent of the claims (84 out of 151) and accepted compensation claims (28 out of 51).

As Meyer Children’s Hospital serves as a reference center for the diagnosis and treatment of rare diseases, we examined the number of cases for each specialty that included subjects with rare diseases. We noted that in neurosurgery, 75% of claims were advanced by patients with rare diseases, while in otorhinolaryngology, the claim rates were 54.5% and 40% in Anesthesia and Intensive care, and 22% in pediatric surgery.

### 3.7. Type of Error

The main types of issues are commissive in surgical specialties, while in medical specialties, they are omissions in diagnosis. The former problems are likely linked to technical issues, and the latter issues are likely linked to diagnostic difficulties.

Therapeutic mistakes, health-related infections, and the inappropriate use of medical devices are rare issues.

**Figure 11:**
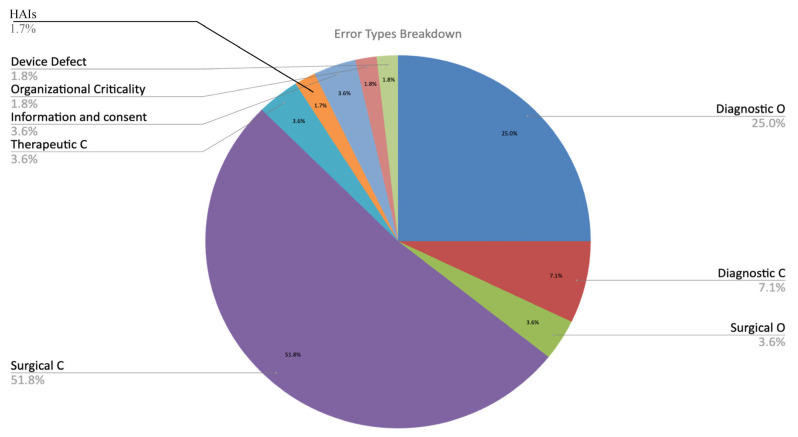
Medical errors categories (Legend: Diagnostic O = diagnostic omissive error in deep blue; Diagnostic C = diagnostic commissive error in red; Surgical O = surgical omissive error in green; Surgical C = surgical commissive error in purple; Therapeutic C = therapeutic commissive error in blue, HAIs = Healthcare-associated infections in orange).

In summary, the significant factors that resulted in the acknowledgment of the Meyer Children’s Hospital’s accountability are classified into various error categories, as illustrated in the graph (Figure 11) below:

Of these 56 claims for which Meyer Children’s Hospital was found liable,

- Forty-two cases were concluded with compensation for damages.
- Six are still pending.
- Seven were unsuccessful.
- One was ultimately dismissed, despite a potential finding of liability on the part of Meyer Children’s Hospital, due to the significant difference between the amount recoverable and the compensation requested.

### 3.8. Type of Damage Referred to in the Complaint

Compensation requests were evaluated by focusing on how damage was described and whether there was a technical consultancy for the party (TC).

We examined whether the claims were vague or detailed regarding the types and amounts of damage. The examination included 158 requests, revealing that 133 claims were very broad, 19 included some specifics about the types of damage and six linked young patient deaths to the Meyer Children’s Hospital. Additionally, the evaluation included the progress of claims and expert opinions. The findings are presented in the table (Table 2).

### 3.9. Definition of the Economic Amount of Compensation

Concerning the purely economic dimension of compensation, it was determined that, pertaining to the 51 accepted claims, the average cost of compensation amounted to EUR 68,312.97, with a minimum of EUR 102 and a maximum of EUR 834,731.

### 3.10. ICPS Results

An assessment of compensation in relation to the severity of the outcomes suffered by the injured parties was then conducted. These results are reported in the following table (Table 3).

## 4. Discussion

This case study analysis of Meyer Children’s Hospital reveals important litigation patterns, although the results lack the strength of inferential statistics. The identified trends suggest avenues for future research and provide insights into how claims could be reduced while enhancing patient safety and satisfaction. Considering the sample size (158 claims), the annual claims rate (11.28 cases/year), and the ratio between the number of services and the number of claims (0.0002, which means that for every 10,000 services, two claims occur), it is clear that the data collected positions Meyer Children’s Hospital among the leading hospitals in Tuscany with regard to its claims rate, as confirmed by the Annual Report from the Regional Center for Clinical Risk Management and Patient Safety [26].

The yearly claims rate, which acts as a standard for assessing a facility’s claims frequency, as defined by the Tuscany region, links the total number of claims to the number of hospitalizations occurring within the same year. This indicator highlights that Meyer Children’s Hospital significantly surpasses other hospitals in Tuscany in this regard.

To provide a meaningful benchmark, we compared our data with those of another highly specialized pediatric institution, Bambino Gesù Pediatric Hospital, and acknowledged the disparities in data presentation and certain ambiguities in the published figures.

When analyzing the 2023 statistics regarding hospital beds (176 at Meyer/627 at Bambino Gesù), routine hospitalizations (8787 at Meyer/29,774 at Bambino Gesù), and emergency room visits (40,063 at Meyer/106,208 at Bambino Gesù), the volume ratio between the two facilities was approximately 1:3. This ratio seems to remain consistent when reviewing claims: from 2019 to 2023, Meyer documented 49 compensation claims, while Bambino Gesù recorded 163 claims during the same period [27].

The analysis of the distribution of claimants classified by age and gender revealed significant insights, even in the absence of the relevant comparative literature. The notable proportion of claims involving children under one year old—and, more generally, the 0–3 age group—corresponds with Meyer’s classification as a center of excellence, where the most severe and complex cases requiring early surgical intervention are referred. These conditions are not only associated with more challenging management and are less likely to yield satisfactory outcomes, but they are also associated with increased mortality rates, thereby raising the likelihood of claims. The concentration of cases among 13–14 year olds, particularly in males, is likely linked to the attainment of orthopedic injuries during recreational and sports activities, which are more common in males and experienced an unexpected increase in this age group compared to surrounding years. The overall male predominance, both in total numbers and especially within the 0–3 age category, may be partially explained by the greater risk of trauma and the higher incidence of certain genetically influenced diseases in males; however, these factors do not completely account for the observed difference, suggesting a need for further investigations.

In terms of claims management, our results only partially correspond with the 14th MedMal Report (2012–2022). This report notes an average claim management duration of 2.8 years, with 46.2% of claims being resolved within 2 years and 65.6% within 3 years. Our examination reveals a similar average management time of 2.78 years; however, it highlights that 50% of claims are concluded within 1.84 years, indicating a delay of roughly one year from the incident to the initiation of a claim.

The disparity between the mean and median management durations suggests that Meyer resolves the majority of claims more rapidly than the hospitals referenced in the report, although a small subset of particularly complex cases—requiring more than two procedural steps—result in prolonged resolution times. The effectiveness of the CMC is also evident, as the 56 cases resolved directly were concluded within approximately 19 months. However, no national benchmark is available for comparison, since the Medmal Report defines the closure time of a claim as the period from its opening to its conclusion, without accounting for the number of procedural steps involved or whether the resolution was extrajudicial or judicial. No reliable breakdown by type of resolution exists at the national level. The averages of all claims vary by approximately 1.5 to 5 years depending on the dataset and methodology.

A comparative analysis of rejected and accepted claims shows that rejected claims are generally filed later but resolved more quickly, possibly indicating summary dismissals based on formal criteria. In contrast, accepted claims usually follow extensive and protracted investigations [28].

The MedMal report further notes that accepted claims are processed approximately seven months faster than denied claims. However, this distinction is ambiguous, as the report does not differentiate between outright rejections and so-called “without follow-up” cases—those unresolved owing to claimant inaction but still potentially eligible for compensation. In our cohort, Meyer achieved a compensation acceptance rate of 32% (51 of 158 claims), compared to over 60% in the national report; this difference may be partly attributable to reporting methods and the inclusion/exclusion of ongoing cases, but does not alter the overall trend. Regarding dispute resolution, Meyer settled 66 of 99 cases out of court, regardless of outcome; 56 were resolved directly by the CMC without attempting ADR, increasing to 102 of 138 (74%) when cases later determined to have “no further effect” were included. Seven cases (5%) were resolved under Article 696 bis (preventive technical assessment regulated by the Italian Civil Law), while 14 cases (10%) ended with a court ruling.

No published literature allows for a direct comparison of the frequency of claims by specialty in centers fully comparable to Meyer Children’s Hospital. Some studies address specific pediatric fields or themes, such as anesthesiology, neonatal care, orthopedics, neurosurgery, radiology, otolaryngology, appendicitis management, and emergencies, yet none provide a holistic institutional perspective [15,29,30,31,32,33,34,35].

In comparing our findings with the eight-year Italian survey (2005–2012) conducted by Fanos et al. [15], which indicated that the majority of claims were related to death or permanent impairment, alongside common issues such as surgical complications, infections, dehydration-related problems, and diagnostic errors, we note that at Meyer Children’s Hospital, a smaller percentage of claims pertained to death and severe impairment. Meanwhile, as previously reported, the primary concerns remain in the areas of surgery and therapy.

When comparing the five-year French study (2003–2007) carried out by Najaf-Zadeh et al. [14] and the four-year study conducted in Turkey by Ozdemir et al. [13] with our findings, we observe a minimal percentage of claims related to deceased or severely impaired children, as well as a comparable percentage of diagnostic or treatment errors.

Moreover, when we compare our findings with those presented in the MedMal report, which identifies the eight most pertinent specialties, the distinctiveness of Meyer’s case mix becomes apparent. Neurosurgery is the most common specialty in Meyer’s claims, whereas it ranks fifth nationally, underscoring Meyer’s emphasis on complex, high-risk pathologies. It is noteworthy that only 6 out of 25 neurosurgery cases at Meyer Children’s Hospital resulted in a compensation payout, indicating a field that is susceptible to litigation yet clinically justified. Similar patterns are observed in claims associated with pediatric surgery and medical pediatrics. Conversely, general surgery shows a comparable burden in both contexts. Orthopedic claims, which are the most prevalent on a national level, are less frequent at Meyer Children’s Hospital, likely due to differences in patient demographics, a diminished need for prosthetics, and a lower occurrence of significant fractures. Emergency medicine, which holds the second position nationally, is less prominent at Meyer Children’s Hospital due to variations in case mix, diagnostic approaches for non-verbal patients, and the relative rarity of conditions such as ischemic heart disease. The increase in otolaryngology claims at Meyer Children’s Hospital can be primarily linked to the recent implementation of a specific endoscopic procedure.

Considering the precision or generality in the formulation of compensation claims, it appears of interest that generic claims, compared to those submitted with specific terms, are associated with a higher frequency of cases closed without further action (26% vs. 15%) and a lower frequency of accepted cases (30% vs. 47%); meanwhile, this difference does not appear to be as relevant regarding rejected cases (30% vs. 26%). However, these data lack robustness given the extremely small number of cases in which a compensation claim was submitted in specific terms (only 19 cases). Nonetheless, this may be indicative of a tendency to initiate “exploratory” litigation—claims not supported by even a preliminary assessment of feasibility and, as a result, unlikely to be accepted or abandoned by the claimants themselves during the process.

An alternative explanation may be the insufficient specialization of the technical consultations provided by the parties involved, coupled with the frequent occurrence of highly complex cases involving multi-pathological disorders, which necessitates advanced skills in the pursuit of damage claims.

Regarding the financial implications of compensation, the average cost per claim recorded at Meyer (EUR 68,312.97) is significantly lower than the scenario reported by Marsh (EUR 116,266.00). This statistic becomes even more significant when we consider that the report highlights the claims with the highest economic impact in the following areas: pediatrics/neurology/NICU (EUR 277,723.00); neurosurgery (EUR 198,100.00); general surgery (EUR 117,647.00); and emergency room (EUR 97,632). This observation may be elucidated by the notion that the heightened severity of the medical conditions treated at Meyer Children’s Hospital leads to lower compensation in the event of an error, owing to the importance of the pre-existing condition.

This hypothesis is, however, challenged by the ICPS outcomes, which show that 12 of the 51 compensated claims involved either death or severe harm, with more than half displaying only mild or no adverse outcomes. A comparison with a large study from a non-pediatric hospital in Rome revealed similar proportions of mild/null cases, a higher incidence of moderate-severity claims at Meyer, and a substantially lower death rate (5% at Meyer vs. 21% in Rome) [36].

The significant prevalence of rare diseases within neurosurgical claims (75%) is noteworthy, aligning with Meyer’s clinical profile; however, the data available for other specialties and for a comprehensive trend analysis are still insufficient to draw definitive conclusions. The rate of cases rejected even after external consultation (around 50%) indicates that there is room for improvement in the effectiveness of CMC’s out-of-court management strategies. Research on litigation at Meyer Hospital from 2010 to 2023 shows the remarkable outcomes, in absolute terms, that the hospital has achieved in handling litigation. The low number of claims and the development of these claims suggest that Meyer Children’s Hospital upholds a high standard of care and is skilled at identifying critical issues as they emerge, thus enabling the implementation of appropriate corrective and preventive actions. However, prolonged processing times, a considerable number of denials, and numerous pending cases highlight areas that require improvement—often linked to the challenges claimants encounter in locating qualified consultants for intricate cases, which can hinder both the submission and resolution of claims. Extended processing times may also stem from the complexities involved in assessing injuries in infants and children. Assessment approaches must account for the developmental factors that influence how children and adolescents experience and express trauma. The literature emphasizes that trauma assessment in young populations faces unique challenges, including derivation and validation problems, developmental influences, and the applicability of current diagnostic classifications. Age-specific considerations are crucial, with distinctions made between measures validated for children (0–12 years) and adolescents (12–18 years).

The assessment of trauma in children and adolescents is complicated by ongoing developmental processes that affect how symptoms manifest and evolve over time. Young people may present with different symptom patterns compared to adults, requiring specialized knowledge and assessment approaches [37,38].

The effective operations of the CMC, as previous reported in another Italian study [39], have also come to light, leading to the resolution of the vast majority of claims out of court. Other notable findings include the very low prevalence of claims related to informed consent or communication, with only two cases in the entire sample centering on these issues. This favorable record highlights the efficacy of Meyer’s strategy in prioritizing holistic, age-appropriate communication with both patients and families; this is a particularly important approach in pediatrics, where paternalism is historically entrenched [40,41,42].

Comparisons with the existing literature pose challenges due to the absence of directly comparable institutional studies [43,44,45,46]. However, the prolonged claim management durations noted at Meyer, while not currently a critical issue, indicate a potential area for enhancement. Reducing this latency may mitigate the risk of litigation instigated by impatient claimants and improve reserve planning. By internalizing essential forensic and loss adjustment functions—presently outsourced—investigations could be accelerated, rapid responses could be facilitated, staff training could be enhanced, and further tools for risk containment and monitoring could be made available.

## 5. Conclusions

In conclusion, despite the limitations of the descriptive statistics employed, our data provide a reliable foundation for future comprehensive studies and continuous monitoring, both within Meyer and in comparison with other similarly specialized centers. The consistently low rate of litigation in a pediatric hospital, the quantification of compensation, and the time it takes the CMC to process claims in relation to its agreement with the judicial evaluation of cases, following CMC-managed settlements, reinforces the perspective that direct claims management by hospitals effectively balances patient rights, provider protection, and institutional accountability, yielding tangible economic and organizational advantages over conventional insurance management. The minimal ratio of compensation requests in relation to the total number of services provided indicates, based on the claim metric utilized, an exceptionally high standard of care, which aligns with other national pediatric institutions. This finding suggests that the approach used in the treatment of pediatric patients could serve as a beneficial model for adult hospitals. These findings advocate for the wider implementation of such a model as a significant advancement in the management of medical malpractice.

## Figures and Tables

**Table 1:** Variables and parameters used in the study.

Variables	Parameters
**Unique identification code of the claim**	Implemented to ensure traceability within the database
**Age and sex of the patient**	Not applicable
**Event date**	Day, month and year
**Method of admission to the hospital**	Ordinary/urgent/emergency
**Type of setting**	Emergency department, hospitalization (which encompasses short-term hospitalizations, such as day and week hospitals), outpatient services, consultation, teleconsultation, and laboratory services
**Specialist area associated with the incident that led to the compensation request**	Not applicable
**Nature of the issue for which compensation is sought**	Surgical errors (concerned invasive procedures, including endoscopic treatments and outpatient surgical procedures). Therapeutic errors involved the administration of drugs or other non-invasive forms of therapy. Diagnostic errors (pertaining to the recognition of a pathological condition, whether through laboratory or instrumental examinations, or by direct patient observation). For these three types of errors, the subcategories “commissive” (when the error consisted in performing an action that should not have been performed) and “omissive” (when the error involved omitting an action that should have been performed) were adopted. Information and consent (where it was not possible to demonstrate the acquisition of valid consent following comprehensive and proper information). Organizational deficiencies (when the error resulted from inadequacies in internal procedures within the institution, such as sterilization or transport procedures). Device defects (including cases in which the defect could potentially be attributed to the manufacturer) were also categorized.
**Date of formulation of the first compensation request**	Day, month, and year
**Date of closure of the litigation**	Day, month, and year
**Type of damage referred to in the complaint**	Generic, specific, or death
**Technical consultancy on behalf of the party**	Not applicable
**Involvement of third parties and agreements with other hospitals**	Not applicable
**Medico-legal evaluation of the damage**	CMC evaluation and recurrence of judicial technical consultancy
**Path of the claim**	The type and number of judicial and extrajudicial steps undertaken were considered, including the sequence in which these steps occurred
**Claim outcome**	Accepted if, at the conclusion of the litigation process, any sum of compensation is granted (blue in the columns); Rejected if, at the conclusion of the litigation process, no compensation is granted (red in the columns); In progress if the case is actively developing, irrespective of the stage attained and any significant inclination towards acceptance or rejection (purple in the columns); Without a follow-up if, despite repeated reminders, the claimant failed to respond to Meyer’s requests for documentation, for undergoing a medicolegal examination, or for providing clarification. This lack of cooperation prevents Meyer from proceeding further with the processing of the case. The time required for the CMC to classify a case as closed without further action is generally 18 months (green in the columns).
**Economic amount of the compensation definition**	Amount in euros
**Recurrence of rare diseases**	All subjects were considered to have a rare disease if there was documentary evidence of them being affected by pathological conditions, or variants thereof, whose recurrence in the literature is reliably attested to be less than or equal to 1 in 2000. Subjects were classified as affected even if the rare disease in question was not related to the issue that led to the compensation claim.
**ICPS results**	The damage resulting from such liability has been categorized according to the International Classification of Patient Safety. This classification identifies the following levels: - None—Patient outcome is not symptomatic, no symptoms are detected, and no treatment is required. - Mild—Patient outcome is symptomatic, intervention is required (e g., additional operative procedure; additional therapeutic treatment), an increased length of stay is required, or permanent or long-term harm or loss of function is caused. - Moderate—Patient outcome is symptomatic, intervention is required (e g., additional operative procedure; additional therapeutic treatment), an increased length of stay is required, or permanent or long-term harm or loss of function is caused. - Severe—Patient outcome is symptomatic, life-saving intervention or major surgical/medical intervention is required, and a shortened life expectancy, major permanent or long-term harm, or loss of function is caused. - Death—When balancing the probabilities, death was caused or hastened in the short term by the incident.

Bold formatting to enhance the variables of the study.

**Table 2:** Type of injury requested.

INJURY SPECIFICATION	N° OF CLAIMS	ACCEPTED	REJECTED	WITHOUT FOLLOW-UP	IN PROGRESS
GENERIC	133	41	40	35	17
With TC	48	18	14	5	11
Without TC	85	23	26	30	6
SPECIFIC	19	9	5	3	2
With TC	16	7	5	2	2
Without TC	3	2	0	1	0
DEATH	6	1	3	1	1
With TC	3	1	1	0	1
Without TC	3	0	2	1	0

**Table 3:** Amount of compensation in euros based on ICPS results.

ICPS Results	N°	Median Compensation	Mean Compensation	Min Compensation	Max Compensation
None	3	2200	5767.33	102	15,000
Mild	23	3371.75	7770.66	553.07	60,000
Moderate	13	16,085.95	20,638.66	8000	73,391.5
Severe	9	120,000	231,447.93	73,247.2	834,731
Death	3	321,500	264,782.15	54,954.45	417,892

## Data Availability

The original and complete datasets presented in this article are not readily available because they include personal data of patients protected by GDPR. Requests to access the datasets should be directed to Meyer Children Hospital, Florence.
